# The anti-apoptotic BAG3 protein is expressed in lung carcinomas and regulates small cell lung carcinoma (SCLC) tumor growth

**DOI:** 10.18632/oncotarget.2261

**Published:** 2014-07-25

**Authors:** Gennaro Chiappetta, Anna Basile, Antonio Barbieri, Antonia Falco, Alessandra Rosati, Michelina Festa, Rosa Pasquinelli, Daniela Califano, Giuseppe Palma, Raffaele Costanzo, Daniela Barcaroli, Mario Capunzo, Renato Franco, Gaetano Rocco, Maria Pascale, Maria Caterina Turco, Vincenzo De Laurenzi, Claudio Arra

**Affiliations:** ^1^ Functional Genomic Unit, Istituto Nazionale per lo Studio e la Cura dei Tumori “Fondazione Giovanni Pascale”, IRCCS, Naples, Italy; ^2^ Department of Pharmacy, University of Salerno, Fisciano, Italy; ^3^ BIOUNIVERSA S.r.l., University of Salerno, Fisciano, Italy; ^4^ Animal Facility Unit, Istituto Nazionale per lo Studio e la Cura dei Tumori “Fondazione Giovanni Pascale”, IRCCS, Naples, Italy; ^5^ Medical Oncology Unit, Thoraco-Pulmonary Department, Istituto Nazionale per lo Studio e la Cura dei Tumori “Fondazione Giovanni Pascale”, IRCCS, Naples, Italy; ^6^ Department of Experimental and Clinical Sciences, University G. D’Annunzio and Fondazione G. D’Annunzio, Ce.S.I., Chieti, Italy; ^7^ Department of Medicine and Surgery, University of Salerno, Fisciano, Italy; ^8^ Pathology Unit, Istituto Nazionale per lo Studio e la Cura dei Tumori “Fondazione Giovanni Pascale”, IRCCS, Naples, Italy; ^9^ Thoracic Surgery Unit, Thoraco-Pulmonary Department, Istituto Nazionale per lo Studio e la Cura dei Tumori “Fondazione Giovanni Pascale”, IRCCS, Naples, Italy

**Keywords:** BAG3, SCLC, NSCLC, tumor growth, apoptosis

## Abstract

BAG3, member the HSP70 co-chaperones family, has been shown to play a relevant role in the survival, growth and invasiveness of different tumor types. In this study, we investigate the expression of BAG3 in 66 specimens from different lung tumors and the role of this protein in small cell lung cancer (SCLC) tumor growth. Normal lung tissue did not express BAG3 while we detected the expression of BAG3 by immunohistochemistry in all the 13 squamous cell carcinomas, 13 adenocarcinomas and 4 large cell carcinomas. Furthermore, we detected BAG3 expression in 22 of the 36 SCLCs analyzed. The role on SCLC cell survival was determined by down-regulating BAG3 levels in two human SCLC cell lines, i.e. H69 and H446, *in vitro* and measuring cisplatin induced apoptosis. Indeed down-regulation of BAG3 determines increased cell death and sensitizes cells to cisplatin treatment. The effect of BAG3 down-regulation on tumor growth was also investigated in an *in vivo* xenograft model by treating mice with an adenovirus expressing a specific bag3 siRNA. Treatment with bag3 siRNA-Ad significantly reduced tumor growth and improved animal survival. In conclusion we show that a subset of SCLCs over express BAG3 that exerts an anti-apoptotic effect resulting in resistance to chemotherapy.

## INTRODUCTION

Lung cancer is the leading cause of cancer-related deaths worldwide, accounting for over 200,000 new cases and over 160,000 deaths per year in the United States, the most aggressive form being small cell lung cancer (SCLC) that accounts for >12% of all lung cancer diagnoses. In fact, SCLC patients have a median survival of 15-20 months and 5-year survival below 15% [1]. Treatment of SCLC is challenging, with a short disease-free survival after 1st line therapy [1, 2]. It is therefore of uttermost importance to gain a deeper understanding of the molecular events involved in SCLC tumorigenesis and progression in order to identify novel potential targets for therapy.

Bcl2-associated athanogene 3 (BAG3) belongs to a family of co-chaperones known to interact with the ATPase domain of the heat shock protein 70 (Hsp70) through the structural domain known as BAG domain (110-124 amino acids). In addition, BAG3 contains a WW domain, a proline-rich region (PXXP), and two conserved IPV (Ile-Pro-Val) motifs, that can also mediate binding to other proteins. While BAG3 can be induced in response to stress in cells of various origin it is constitutively expressed only in a few, including skeletal muscle and the heart. Importantly it is has also been shown to be constitutive in several primary tumors and tumor cell lines (pancreatic cancer, melanoma, leukemias, and others) [3, 4] and has been shown to play an important role in tumor biology [3, 4, 5, 6, 7, 8]. It has been suggested that its role in tumors is due to its anti-apoptotic properties in fact BAG3 has been shown to protect cells from death through a number of mechanisms that in general involve interaction with apoptosis- regulating proteins, including the IKK gamma subunit of the NF-κB- activating complex IKK [5], Bax [9], BRAF [10] and others [3].

Here we investigated BAG3 expression in SCLCs and its role in tumorigenesis in a xenograft mouse model. Our data suggest that indeed BAG3 is a potential target in a subset of tumors that express it.

## RESULTS

### Immunohistochemical analysis of BAG3 expression in human lung neoplastic samples

**Figure 1:**
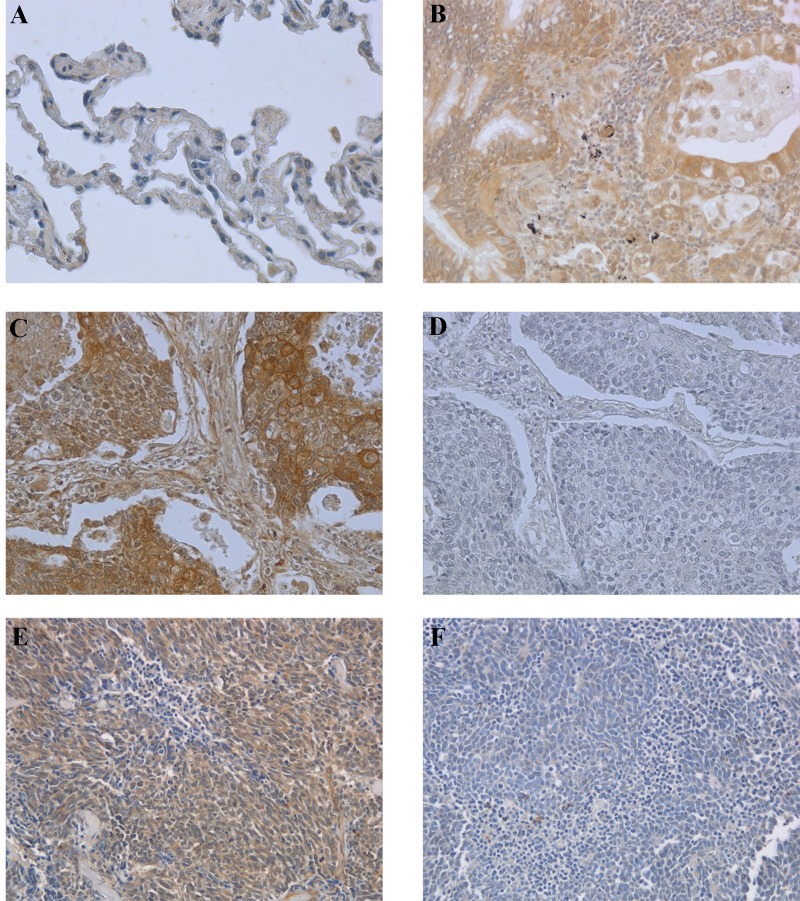
Immunohistochemical analysis of BAG3 expression in normal and neoplastic lung samples Paraffin sections from 33 NSCLCs, 36 SCLCs and 3 normal lung specimens were analyzed by immunohistochemistry using anti-BAG3 rabbit polyclonal antibody. A) Normal lung tissue sample showing no immunoreactivity for BAG3 (200x). B) Lung adenocarcinoma showing BAG3 cytoplasmic staining (200x). C) Squamous cell carcinoma sample showing cytoplasmic BAG3 staining (200x). D) Immunostaining of the same squamous cell carcinoma sample as in panel C stained without the primary antibody (200x). E) SCLC sample showing BAG3 cytoplasmic staining (200x). F) SCLC sample negative for BAG3 protein expression (200x).

We analyzed BAG3 expression in 69 samples (36 small cell lung cancers, 13 squamous cell carcinomas, 13 adenocarcinomas, 4 large cell carcinomas and 3 normal lung samples as control) by immunoistochemistry (IHC), using an anti-BAG3 polyclonal antibody (TOS-2). As expected no BAG3 expression was detected in normal lung while all the NSCLC and 22 out of the 36 SCLC samples were BAG3- positive (Table 1 and Fig.1). Positive tumor samples showed a cytoplasmic staining in the majority of the cells.

**Table 1:** Immunohistochemical analysis of BAG3 expression in normal and pathological human lung tissues

Histological type	N. of total cases analized	BAG3 staining score*			
		0	1	2	3
Normal lung tissue	3	3			
Squamous cell carcinoma	13	0	2	5	6
Adenocarcinoma	13	0	4	7	2
Large cell carcinoma	4	0	1	1	2
Small cell lung cancer	36	14	10	8	4

* Human lung specimens were stained with anti-BAG3 polyclonal antibody. The percentage of malignant cells stained was scored from 0 to 3: 0, no positive cells; 1+,<10% of positive cells; 2+,11-50% of positive cells; 3+, 51–100% of positive cells.

### Effect of BAG3 down-modulation on SCLC cell apoptosis

**Figure 2:**
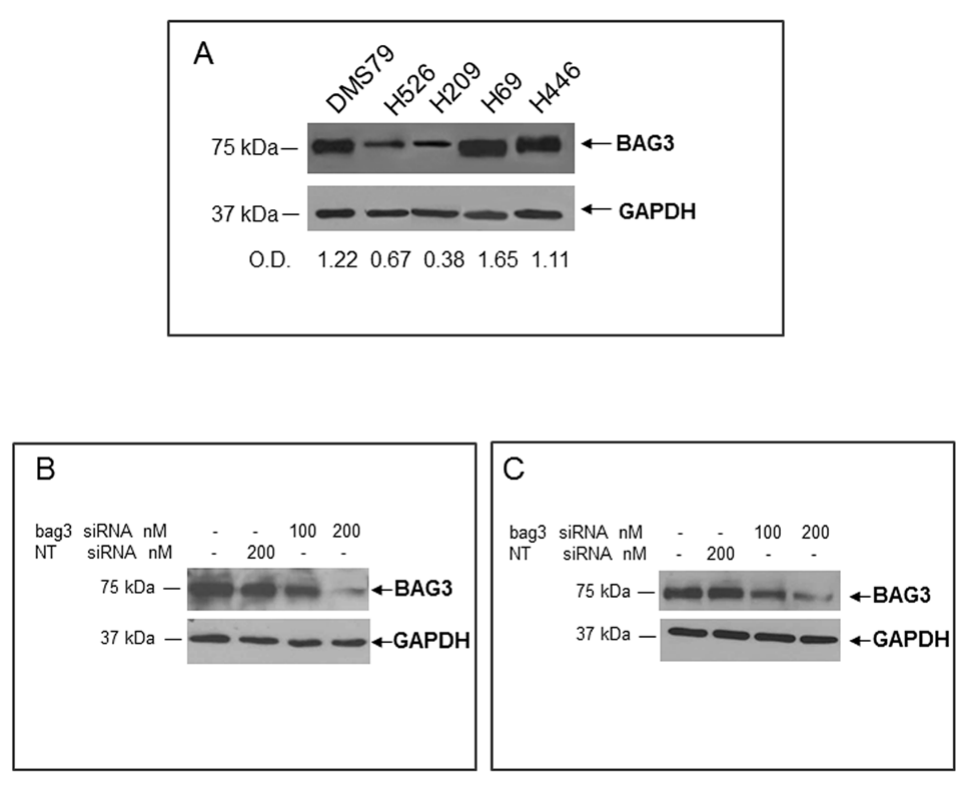
Analysis of BAG3 protein levels in human SCLC cell lines A) Total protein extracts obtained from DMS79, H526, H209, H69 and H446 cell lines were analyzed by western blot using an anti-BAG3 polyclonal antibody. Densitometry data of samples are expressed as fractions of BAG3/GAPDH. H69 cells (B) and H446 cells (C) were transfected with a bag3-specific (bag3 siRNA) (100 and 200 nM) or a NT siRNA (200 nM). After 72 hrs BAG3 protein levels were analyzed by western blot using an anti-BAG3 polyclonal antibody. Anti-GAPDH antibody was used as loading control.

**Figure 3:**
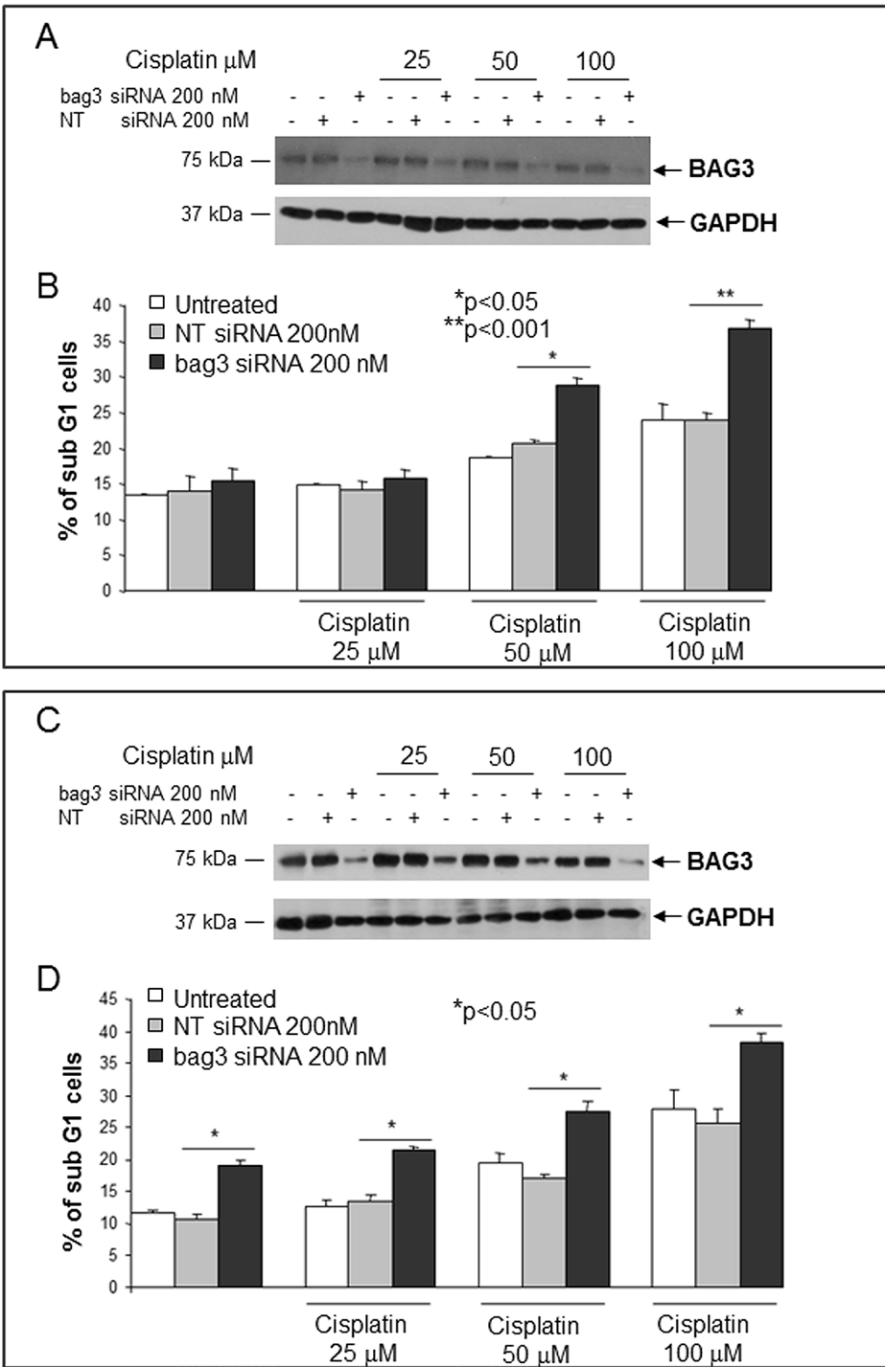
Effect of BAG3 down-regulation on SCLC cell apoptosis H69 (A, B) or H446 (C, D) cells were transfected with bag3 siRNA (200 nM) or with NT siRNA (200 nM) and after 48 hrs treated with different doses of Cisplatin (25, 50, 100 microM) for 48 hrs. Cells were then collected and labeled with propidium iodide and analyzed by flow cytometry (B, D). Total protein extracts of cells treated as described above were analyzed by western blot using an anti-BAG3 polyclonal antibody. Anti-GAPDH antibody was used as loading control (A, C). *p<0.05; **p<0.001.

We then investigated the possibility that in BAG3 positive SCLCs this protein played a pro-survival role as reported for other tumor types [3, 4]. To this end we first evaluated BAG3 expression by western blot in five human SCLC cell lines. As shown in Fig. 2A, H69 and H446 cell lines displayed the highest BAG3 protein levels and were chosen for the subsequent experiments to test if BAG3 silencing sensitized cells to cisplatin treatment. Both cell lines were transfected with a bag3- specific small interfering (si) RNA or with a non targeted (NT) siRNA. As shown in figures 2B and C transfection with 200 nM of Bag3 specific siRNA resulted in silencing of BAG3 in both cell lines. As shown in figure 3B and D silencing of BAG3 results in increased response to cisplatin with an increase in both cell lines of almost 40% of apoptosis after 48 hours of treatment with a 100 μM cisplatin. Furthermore, BAG3 silencing in H446 results also a significant increase of basal apoptosis (Fig. 3D).

These results indicate that down-regulation of BAG3 stimulates apoptosis in SCLC cells *in vitro*.

### Down-regulation of BAG3 reduces in vivo tumor growth and induces apoptosis

**Figure 4:**
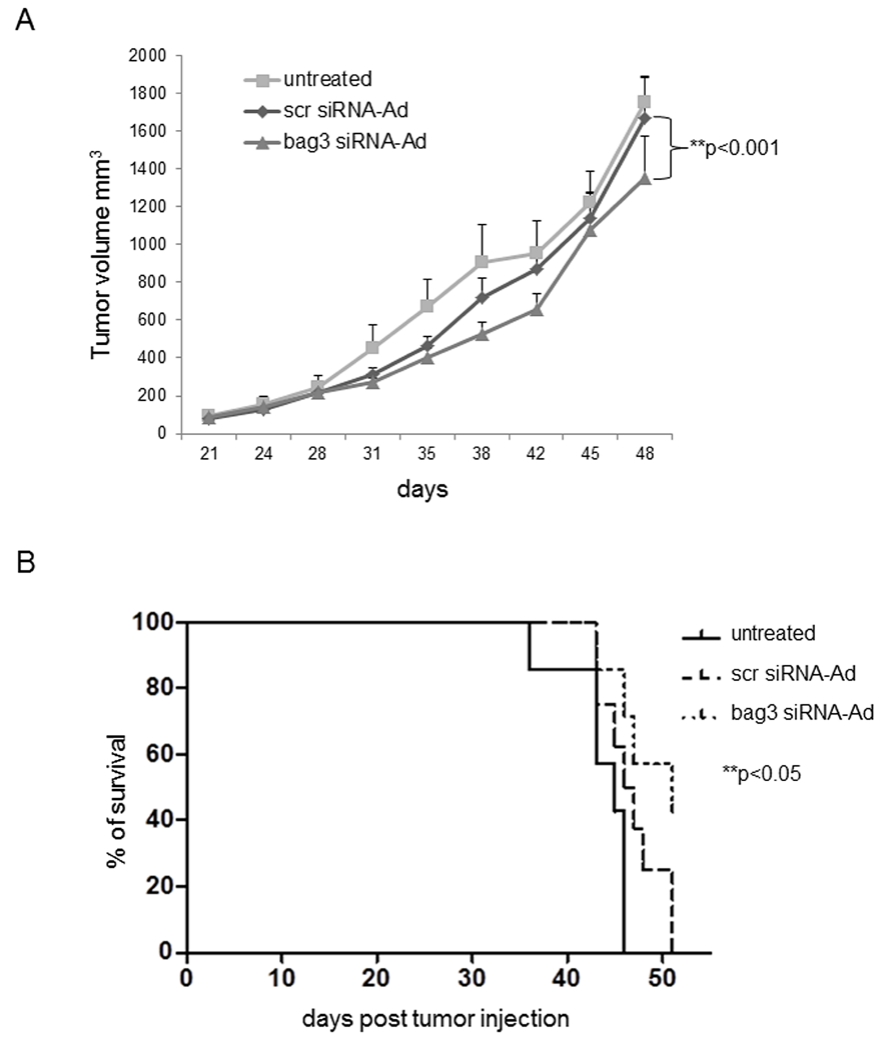
BAG3 down-regulation reduces tumor growth and increases animal survival in a xenograft model H69 cells (3x 10^6^) were injected subcutaneously onto the back of six/eight-week-old female athymic nude-Foxn1nu/nu mice. Two weeks later (100 mm^3^), animals were randomized into three groups (8 animals per group) and control PBS (100μl-untreated), bag3 siRNA-Ad or scrRNA-Ad (10^8^ pfu/100 μl) were injected in the tumors twice a week. A) Tumor size was measured every week using a caliper in bag3 siRNA-Ad, scr siRNA-Ad treated or untreated mice. **p< 0.001. B) Kaplan-Meier analysis of animal survival. *p< 0.05.

**Figure 5:**
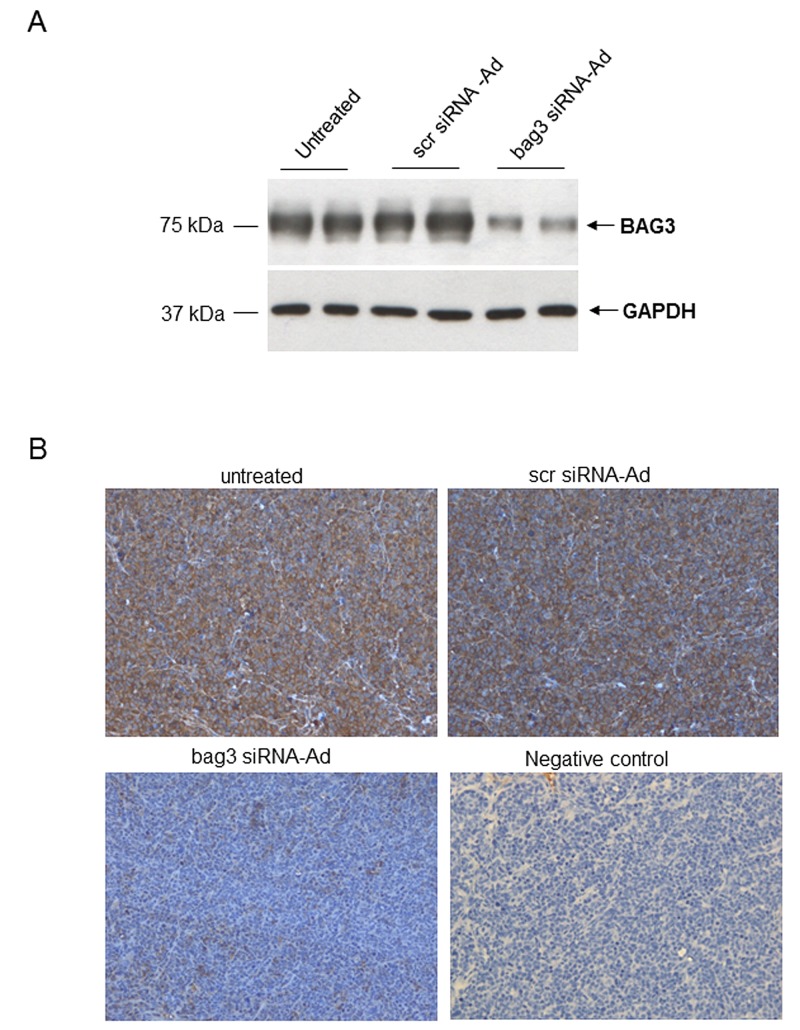
Analysis of BAG3 expression in H69 xenografts A) Western blot analysis of protein extracted from H69 xenografts treated with PBS 100 μl (untreated), scr siRNA-Ad (10^8^ pfu/100μl) or bag3 siRNA-Ad (10^8^ pfu/100μl) by intratumoral injection, twice a week. B) Immunohistochemistry with anti-BAG3 monoclonal of xenograft tumors treated as in A (200x). Negative control: immunostaining of xenografted tumor samples stained without the primary antibody (200x).

Since in vitro data showed that down-regulation of BAG3 induces apoptosis in SCLS cells, we investigated the effects of bag3 siRNA-Ad treatment on tumor growth and apoptosis *in vivo*. As shown in Fig. 4A, we observed that intra-tumoral injection of bag3 siRNA-Ad was able to reduce *in vivo* tumor growth after 44 days of treatment as compared to the scramble-treated (scr siRNA-Ad) and control groups (p<0.001). Accordingly, mice treated with bag3 siRNA-Ad survived longer than control (PBS) and scr siRNA-Ad-treated mice (p<0.05) (Fig. 4B). Western blot and immunohistochemical analysis of tumor samples from xenografts treated for 12 days with bag3 siRNA-Ad specifically confirm a reduction of BAG3 protein levels (Fig. 5 A, B).

**Figure 6:**
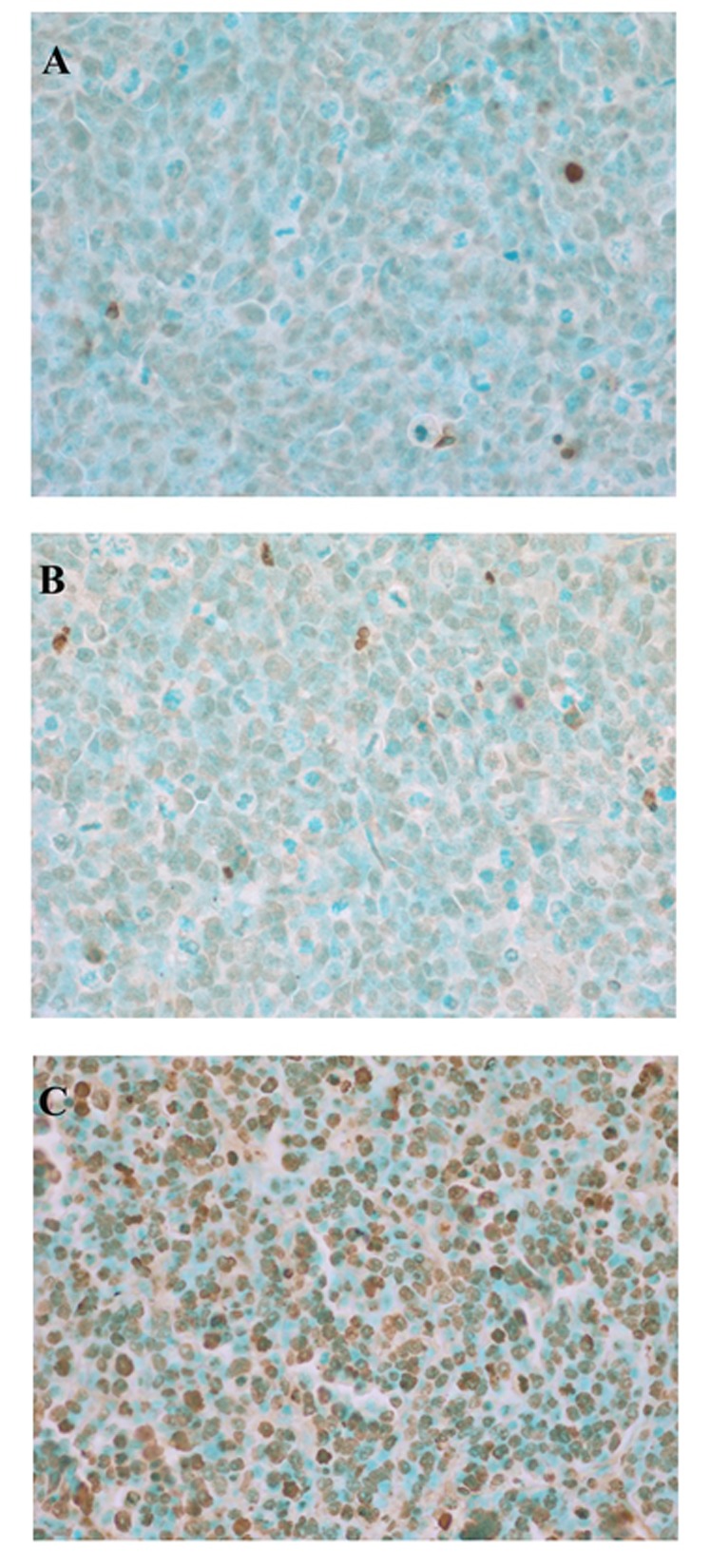
BAG3 down-regulation induces apoptosis in H69-xenografted mice TdT-FragEL staining of samples from H69 xenografts treated with PBS (untreated-A), scr siRNA-Ad (B) or bag3 siRNA-Ad (C) by intratumoral injection, twice a week.

Evaluation of apoptosis using the terminal deoxynucleotidyl transferase (TdT) FragEL™ DNA fragmentation detection kit, an analogue of the TUNEL method, shows that intra-tumoral injection of bag3 siRNA-Ad induced massive apoptotic cell death in tumor cells (Figure 6C).

## DISCUSSION

Molecular classification of lung cancer has allowed targeted chemotherapeutic strategies. The understanding of the role of gene mutations (EGFR, K-RAS and MET), gene fusions (ALK) and rearrangements (ROS-1), has improved the management of non-small-cell lung cancer patients [11]. We suggest that evaluation of BAG3 expression might be a potentially useful classification tool for SCLCs.

BAG3 is an anti-apoptotic protein that has been shown to sustain cell survival in a variety of tumor types [5, 6, 9, 10], and indeed its down-regulation appears to induce cell apoptosis and impair tumor growth also in SCLC cells suggesting that it may represent a novel target for therapy in positive tumours.

The pro-survival role of BAG3 in tumors largely relies on its ability to interact with different partners, some of which are selectively involved in the growth of specific tumor types [4]. The molecular mechanisms and the proteins involved in BAG3 pro-survival function in SCLCs are still unknown and require further studies. Recent reports indicate a relevant role for the MET signaling pathway in SCLC biology [12, 13, 14, 15]. Indeed, Met receptor phosphorylation induced by HGF is associated to the hyperexpression of mesenchymal markers and chemoresistance that result in a poor prognosis in SCLC patients [14, 15]. Furthermore, MET receptor signaling leads to the activation of PI3K–Akt signaling, Ras–MAP kinase cascade, STAT3 and NF-κB activation, promoting cell survival, proliferation and invasiveness [16]. Some of these signaling proteins, including NF-κB factors [5], RAF family proteins [10] and the downstream kinase ERK [17], have been shown to interact with BAG3, that can modulate their levels or activity and are therefore potentially involved in BAG3 mechanism of action in these cells.

Recently, it was demonstrated that BAG3 knockdown induces Epithelial Mesenchymal Transition (EMT) in thyroid cancer cells increasing their metastatic potential [18]. However Xiao H. et al. have opposite results and show that BAG3 overexpression is associated with the increased angiogenesis and invasive ability of hepatocellular carcinoma cells [19]. These different results can possibly be explained by the fact that BAG3 can exert different effects depending on the cellular context and therefore on the different proteins it interacts and regulates [4]. Our next aim will be focused on a more deeper understanding of the role of BAG3 in EMT cellular context in orthotopic animal model of SCLC. It is therefore important to study the effects of altered expression of BAG3 in the different contexts to fully understand its biological role. Moreover it is possible that the effect of BAG3 on growth and survival of the primary tumor cell and on EMT and invasive capacity are independent and rely on different signaling pathway so it is possible that while increased BAG3 favors tumor cell survival and growth it prevents EMT and metastatic spreading. While clearly both effects of BAG3 have to be taken in account when proposing a new therapeutic approach, this goes beyond the scope of this manuscript in which we focused on the role of BAG3 on growth and survival of the primary tumor.

In conclusion, our studies show that increased expression of BAG3 in SCLCs represents an advantage for these cells resulting in reduced cell death and increased resistance to therapy and that evaluating BAG3 expression may represent a potential marker of prognosis and of response to cisplatin therapy. Finally, BAG3 could be a novel target for therapy for the subset of SCLCs that over-express it.

## MATERIALS AND METHODS

### Cell lines

The SCLC cell lines DMS79, H209, H69, H526 and H446 were provided by the American Type Culture Collection and grown in RPMI-1640 medium supplemented with 10% fetal bovine serum (Cambrex Bio Science), 2mM L-glutamine, 10mM Hepes, 1mM sodium pyruvate, 1% penicillin-streptomycin mixture, at 37°C, in a 5% CO2 atmosphere.

### Reagents

The polyclonal (TOS-2) and monoclonal (mAb) (AC-1) antibodies against human BAG3 protein were provided by Biouniversa srl, Italy. Anti- GAPDH mAb was obtained from Santa Cruz Biotechnology Inc. Enhanced chemiluminescence Western blot detection reagents were purchased from Amersham Life Sciences Inc. (Pennsylvania Pittsburgh, USA). Secondary antibodies were purchased from Pierce (Meridian Rd, Rockford, IL USA).

### siRNAs and adenoviral constructs

bag3 siRNA (5′-AAGGUUCAGACCAUCUUGGAA-3′) and non targeted (NT) siRNA (5′-CAGUCGCGUUUGCGACUGG-3′) were synthesized by Dharmacon (La Fayette, CO). Transfectin (Bio-Rad Laboratories, Inc., Hercules CA) was used for cell transfection. bag3 siRNA-Ad and scr siRNA-Ad were made using the BD Adeno-X Expression Systems 2 PT3674-1 (Pr36024) and BD knockout RNAi Systems PT3739 (PR42756) (BD Biosciences-Clontech, Palo Alto, CA) [5].

### Animals

Twentyfour six/eight-week-old female athymic nude-Foxn1^nu/nu^ mice were purchased by Harlan Laboratories S.rl. (S. Pietro al Natisone, Italy). Mice were housed eight per cage and maintained on a 12 hrs light:12 hrs dark cycle (lights on at 7:00 a.m.) in a temperature-controlled room (22 ± 2°C) with food and water ad libitum. The experimental protocols were in compliance with the European Communities Council directive (86/609/EEC). H69 xenografts were produced on the right flank of the mice by subcutaneous injection of 3×10^6^ H69 cells in 150 μl of Hanks’ balanced salt solution. Three weeks after tumor cell injection, mice with tumors of similar size (about 100 mm^3^) were randomized into three groups (8 mice per group) and treated with control PBS (100 μl), bag3 siRNA-Ad or scr siRNA-Ad (10^8^ pfu/100 microl), by peri-tumoral injection, twice a week. Tumor size was measured every three days by digital caliper 2Biol (Besozzo, Varese, Italy) and tumor volumes were calculated according to the formula: V = (a x b2)/2, where a = the largest superficial diameter and b = the smallest superficial diameter. When the tumor burden reached 750 mm^3^ three mice from each group were euthanized to assess the silencing of BAG3 by immunohistochemistry (IHC). All other mice were sacrificed when tumor size reached 1500 mm^3^ in the control group. Differences among the treatment groups were analyzed by ANOVA using statistical software Graph Pad Prism 5.0 (La Jolla, CA). Survival was analyzed by the Kaplan–Meier method, and survival curves were compared by use of the log-rank test [20].

### Immunohistochemistry

#### Patients

The paraffin sections of thirty patients with NSCLC classified according to the degree of differentiation and thirty-six patients with SCLC were selected by the Pathologists of the Istituto Nazionale Tumori, Fondazione G. Pascale, Napoli, Italy.

#### Xenografts

Excised tumors were immediately fixed in 10% neutral buffered formalin and paraffin embedded.

Immunohistochemical staining of human and xenograft tumor sections was performed as follows. Five- to 6-μm-thick paraffin sections from each tumor were deparaffinised and placed in a solution containing 0.3% hydrogen peroxide at room temperature. After blocking, the humans and xenografts slides were incubated overnight at 4°C in a wet chamber with the anti-BAG3 polyclonal antibody TOS-2 (diluition 1:200 in PBS) and anti-BAG3 monoclonal antibody (mAb) AC-1 (diluition 1:100 in PBS) (Biouniversa Srl, Fisciano, Italy), respectively. Immunoreactivity was visualized using a streptavidin-biotin-peroxidase complex according to the manufacturer's instructions, with diaminobenzidine as peroxidase substrate (Dako REAL^TM^ Detection System, Peroxidase/DAB^+^, Rabbit/Mouse). The stained sections were counterstained with hematoxylin. Negative controls were performed by omitting the primary antibodies (data not shown).

### Western blotting

Cells were harvested and lysed in a buffer containing 20 mM HEPES (pH 7.5), 150 mM NaCl, 0.1% Triton (TNN buffer) supplemented with a protease inhibitors cocktail (1 mM phenylmethylsulfonyluoride, 1 mg/ml pepstatin A, 2 mg/ml aprotinin), and subjected to 3 cycles of freeze-and-thawing. Lysates were centrifuged for 20 min at 12 000 rpm and stored at -80°C. Protein amount was determined by Bradford assay (Bio-Rad, Hercules, CA) and 30 μg of total protein were run on 10% SDS-PAGE gels and subjected to electrophoretic transfer to a nitrocellulose membrane. Nitrocellulose blots were blocked with 10% nonfat dry milk in TBST buffer (20 mM Tris-HCl, pH 7.4), 500 mM NaCl and 0.01% Tween, and incubated with primary antibody in TBST containing 5% nonfat dry milk overnight at 4°C. Immunoreactivity was detected by sequential incubation with horseradish peroxidase-conjugated secondary antibody and ECL reagents. Scanning densitometry of the bands was performed with image scanning software (SnapScan 1212; Agfa-Gevaert, Mortsel, Belgium). The area under the curve related to each band was determined using Gimp version 2.6 software (http://www.gimp.org). Background was subtracted from the calculated values.

### Apoptosis evaluation

Cells transfected with bag3- specific or NT siRNA (48 hours) were incubated with or without cisplatin at the indicated concentrations. After 48 hour, cells were harvested and then incubated with a propidium iodide (PI) solution (0.1% sodium citrate, 0.1 % Triton X-100 and 50 μg/ml of PI), for 30 min at 4°C. Apoptosis was quantified as the proportion of cells with hypodiploid DNA (sub G0-G1 peak) using flow cytometry (FACScan Becton Dickinson). A minimum of 5000 events were recorded and analyzed. Cellular debris was excluded from analysis by raising the forward threshold. All measurements were performed using the same instrument settings. Significance between the groups was calculated by unpaired Student's t test.

Apoptosis on the FFPE (Formalin-Fixed, Paraffin-Embedded tissues) sections from isolated tumor tissues of xenograft, was detected using a TdT-FragEL DNA Fragmentation detection kit (Calbiochem) according to the manufacturer's guidelines.

### Statistical analysis

Results are expressed as means of duplicates ± SD obtained from three independent experiments. Data were analyzed by Student's t-test using GraphPad Prism statistical software (La Jolla, CA).

Significance was evaluated according to the scale: *P<0.05 (significant); **P<0.01 (very significant); ***P<0.001 (highly significant).
